# The Effect of Body Mass Index on Acute Cardiometabolic Responses to Graded Exercise Testing in Children: A Narrative Review

**DOI:** 10.3390/sports6040103

**Published:** 2018-09-20

**Authors:** Pantelis T. Nikolaidis, Eleni Kintziou, Georgios Georgoudis, José Afonso, Rodrigo L. Vancini, Beat Knechtle

**Affiliations:** 1 Exercise Physiology Laboratory, 18450 Nikaia, Greece; pademil@hotmail.com; 2 School of Health Sciences, University of West Attica, 12243 Egaleo, Greece; ekintziou@teiath.gr (E.K.); gg@hol.gr (G.G.); 3 Faculty of Sport, University of Porto, 4200-450 Porto, Portugal; jafonsovolei@hotmail.com; 4 Center of Physical Education and Sport, Federal University of Espírito Santo, 29075-910 Vitória, Brazil; rodrigoluizvancini@gmail.com; 5 Institute of Primary Care, University of Zurich, 9001 Zurich, Switzerland

**Keywords:** body fat, cardiac rate, cycling, exercise intensity, heart rate, metabolism, overweight, respiratory quotient, resting metabolic rate

## Abstract

Although the beneficial role of exercise for health is widely recognized, it is not clear to what extent the acute physiological responses (e.g., heart rate (HR) and oxygen uptake (VO_2_)) to a graded exercise test are influenced by nutritional status (i.e., overweight vs. normal-weight). Therefore, the main objectives of the present narrative review were to examine the effect of nutritional status on acute HR, and VO_2_ responses of children to exercise testing. For this purpose, we examined existing literature using PubMed, ISI, Scopus, and Google Scholar search engines. Compared with their normal-body mass index (BMI) peers, a trend of higher HR_rest_, higher HR during submaximal exercise testing, and lower HR_max_ was observed among overweight and obese children (according to BMI). Independent from exercise mode (walking, running, cycling, or stepping), exercise testing was metabolically more demanding (i.e., higher VO_2_) for obese and overweight children than for their normal-weight peers. Considering these cardiometabolic differences according to BMI in children might help exercise specialists to evaluate the outcome of a graded exercise test (GXT) (e.g., VO_2max_, HR_max_) and to prescribe optimal exercise intensity in the context of development of exercise programs for the management of body mass.

## 1. Introduction

It has been reported that more than half of adults in the USA are either overweight or obese, and that high levels of body mass (BM) or body fat percentage (BF) are associated with increased risk for numerous diseases [1]. High rates of combined overweight and obesity have also been recorded for children and adolescents, e.g., 20–25% in Latin America [2], 40% in southern Europe, and 10% in northern Europe [3]. In addition, obesity has been shown to be associated with increased chance of mortality [4], whereas overweight has been linked to diseases such as hypertension, hypercholesterolemia, and diabetes [5,6]. Overweight and obesity did not only have implications for health, but also had an important financial impact on increasing health care expenditures [7].

From a physiological point of view, an increased BM might result from an excess of energy availability, which in turn might result from either increased energy intake, decreased energy expenditure, or a combination of both. Consequently, most of the research concerning the management of overweight and obesity focused on altering either nutrition intake and/or exercise levels [8]. A review reported that interventions using both exercise and diet resulted in greater BM reduction than diet alone, and that the magnitude of BM reduction depended on exercise intensity, i.e., the higher the exercise intensity, the greater the BM reduction [8].

Thus, information on exercise intensity is crucial to develop efficient aerobic exercise interventions for overweight and obese children. Exercise intensity might be measured by oxygen consumption (VO_2_), metabolic equivalents (METs), lactate concentration, rate of perceived exertion, and heart rate (HR) [9] during a graded exercise test (GXT). When HR is used to prescribe exercise intensity, training zones should be set taking into account resting (HR_rest_) and maximal values of HR (HR_max_), e.g., the Karvonen method [10], in which exercise intensity is expressed as a percentage of HR reserve. An alternative might be simply to express HR as a percentage of HR_max_, which is an easy method to administer, but its weakness is in describing low intensities (e.g., 20% or 30%).

Despite the importance of exercise intensity in intervention programs for overweight and obese children, and the acknowledgment of HR as a measure of exercise intensity, limited data existed concerning acute HR responses to exercise in these population groups. It might be of great practical value to be aware of differences in acute HR responses of these groups compared to their normal-weight counterparts, because this information would assumedly contribute to a better prescription of exercise intensity and subsequently better development of exercise interventions to manage overweight and obesity. For instance, potential differences in HR_max_ by BMI (body mass index) during a GXT might influence the indirect criteria used to assess the attainment of VO_2max_.

### 1.1. Nutritional Status and Body Mass Index

Nutritional status reflects the energy balance, i.e., the relationship between energy intake (nutrition) and energy expenditure (basal metabolic rate, physical activity, and thermic effect of meals) [11]. Assessing BM for a given height (e.g., BMI) has been widely used as a measure of nutritional status. BMI has been used to classify adults as underweight (<18.5 kg·m^−2^), normal-weight (18.5–25 kg·m^−2^), overweight (25–30 kg·m^−2^), and obese (>30 kg·m^−2^) [12,13]. According to this classification, BMI might be evaluated in children using centile curves that at age 18 years pass through the cut-off points used for adults [14,15]. However, there is not a unique definition for children. One possibility is to define those children with BMI higher than 85% as overweight [16], and those with BMI lower than the 10th percentile as lean [17]. Another study, which also used percentiles, defined those children with BMI higher than 95% as overweight [18], whereas Pate and colleagues [19] defined those with BMI lower than 85% as normal-weight, between 85% and 95% as being at risk for overweight, and those with BMI higher than 95% as overweight. On the contrary, those with BMI higher than 95% were defined as obese in other studies [20,21]. Based on percentiles, Reybrouck and colleagues [22] defined obesity as having a BM higher than 90% of the normal values when expressed on a BM for height diagram. Also, Sigmund and colleagues [23], and Govindan and colleagues [24] defined those with a BMI higher than 85% and 97% as overweight and obese, respectively. With regards to body composition, BMI does not provide information whether an excess of BM is due to BF or fat-free mass (FFM). Nevertheless, BMI is used as a proxy measure of BF due to their large correlation [25]. 

### 1.2. Exercise Mode, Intensity, and Protocols

Testing aerobic capacity usually takes place either in a laboratory setting or in the field. In a laboratory, exercise testing is performed on calibrated equipment, which might be a treadmill [22,26,27,28,29], a cycle ergometer [30,31,32], or a step [33,34,35,36], whereas in field testing, participants run a certain distance as fast as they can, e.g., 1 mile run/walk [37], or try to cover the longest distance within a given time, e.g., 12 min run/walk [18], or run with an incremental speed as much as they can, e.g., 20 m shuttle run test [17]. The tests conducted in field—where the environmental conditions (e.g., temperature, humidity, wind, and noise) vary and may interfere with the outcome—are less valid and reliable than those conducted in a laboratory [38,39,40].

Laboratory testing typically includes either maximal or submaximal protocols. An example of maximal protocol is the Bruce protocol [21], which is performed on a treadmill using incremental speed and incline. Maximal tests are also performed on a cycle ergometer [41,42]. On the contrary, submaximal testing does not require maximal effort; rather participants perform at given intensities, like running against 4, 5.6, and 8 km·h^−1^ on a treadmill [16,26]. An example of a submaximal test using a step (YMCA protocol) includes stepping up and down on a 30 cm step bench for 3 min, in which HR is recorded with a stethoscope during the first minute of recovery after the test [24,35]. Another example of a similar protocol is the Queen’s College step test, which includes stepping up and down for 3 min with the same frequency as in YMCA protocol (24 ascends·min^−1^), but using a higher step bench (41.3 cm) [33,34]. Pate and colleagues used a submaximal protocol on a treadmill consisting of two 3-min stages of different intensities.

It should be highlighted that the exercise mode might influence the differences in cardiometabolic responses between normal- and overweight participants in the studies under examination, because the body is supported on a cycle ergometer, in contrast to a treadmill or step, and this might attenuate the effect of BM. In this line, a study on a cycle ergometer reported similar cardiometabolic response to exercise between normal- and overweight children [30]. Furthermore, it has been shown that at similar levels of HR, obese adolescents had had higher VO_2_, energy expenditure, and fat oxidation during exercise on a treadmill than on a cycle ergometer [43]. Another consideration in exercise testing is the inclusion of detailed standardized procedures in the protocols with regards to aspects such as familiarization with the testing procedures, guidelines about nutrition and rest prior to testing, as well as for recommended time-of-the-day for conducting the tests (to prevent diurnal variation) [44].

### 1.3. Maximal Oxygen Uptake and Body Composition

VO_2max_ has been considered as the golden criterion measure of aerobic capacity, which includes the recording of VO_2_ during a GXT usually on a treadmill or a cycle ergometer [45]. In addition to the standard measurement unit (L·min^−1^), i.e., how much oxygen is transferred and consumed in the end of a maximal test, this measure has also been expressed relative to BM unit (mL·kg^−1^·min^−1^) in order to reflect the effect of body size on aerobic capacity [46]. Thus, caution is needed when examining studies on VO_2max_ to consider which measurement unit is being implemented (L·min^−1^ or mL·kg^−1^·min^−1^).

The use of relative units for VO_2_ depends on the applied model (atomic, molecular, cellular, tissue-system, and whole body) of body composition [47]. According to this classification of body composition, VO_2_ is scaled for BM when the whole body is considered. In addition to the traditional relative unit (mL·min^−1^·kg^−1^), another option is allometric scaling [48] suggesting a different consideration of BM, e.g., mL·min^−1^·kg^−0.67^, mL·min^−1^·kg^−0.71^, or mL·min^−1^·kg^−0.75^ instead of mL·min^−1^·kg^−1^ [46,49]. The rationale for the use of allometric scaling is that larger animals increase oxygen transport by having bigger hearts (stroke volume), whereas smaller animals achieve the same effect by increasing their heart rate to very high levels, as it has been indicated by the comparative physiology of animals of different sizes [50]. Moreover, VO_2_ can be scaled for FFM when the tissue-system level in the body composition classification is considered [51].

While the importance of exercise testing for the development of optimal training interventions for BM management is undisputable, there is no comprehensive review regarding differences in cardiometabolic responses by BMI groups during a GXT. Knowledge on such differences could be used by practitioners to design cohort-specific exercise testing protocols. Subsequently, data from these tests could be taken to deduce cardiorespiratory training zones. Therefore, the main aim of the present study was to examine the existing body of literature concerning cross-sectional studies that compared acute HR and VO_2_ responses to exercise during a GXT in groups differing for BMI. A further aim was to study differences according to BMI for other practically relevant cardiorespiratory parameters, such as HR_rest_, resting metabolic rate (RMR), and the respiratory quotient (RQ). Moreover, methodological issues concerning the exercise stimulus used in existing literature, such as exercise intensity (e.g., rest, submaximal, maximal, and recovery) and mode (e.g., running and cycling), were examined.

## 2. Methods

For the purpose of the present narrative review, we thoroughly examined existing literature using Scopus, PubMed, Google Scholar, and Clarivate Analytics search engines in December 2017. Keywords and syntaxes for the search engines were: (“heart rate” OR “oxygen uptake” OR “resting metabolic rate” OR “respiratory quotient”) AND (“body mass index” OR “nutritional status” OR normal-weight OR overweight OR obese) AND (children OR adolescents) NOT (adult OR women OR men). Inclusion criteria comprised studies (*n* = 71) that were published in English only. In addition, studies were eligible for inclusion if they reported data on age, sex, number of participants, BMI, exercise protocol, and HR, and use at least two groups differing in BMI for the purpose of cross-sectional design [52]. Any study that lacked one of the abovementioned elements was excluded from further analysis.

## 3. Results and Discussion

Our search identified 14 studies that applied cardiorespiratory exercise testing in children aged 6–18 years. Most studies (*n* = 8) were conducted in pre-pubertals and included both sexes. Unfortunately, these studies did not separate their findings according to sex. The remaining (*n* = 6) studies examined adolescents and reported sex-specific findings. Sample sizes ranged from 40 to 182 and whenever possible participants were classified in two groups (i.e., normal-weight versus overweight, normal-weight versus obese, or non-obese versus obese).

### 3.1. Acute Oxygen Uptake Responses to Exercise Testing

**Figure 1:**
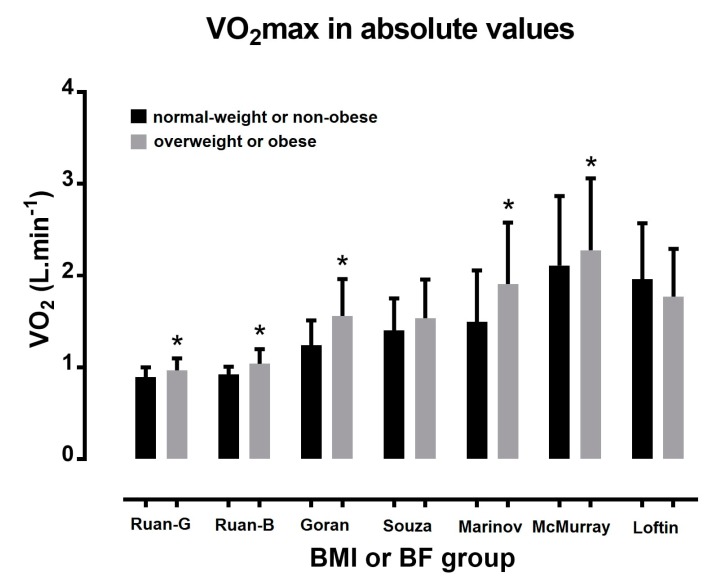
Maximal oxygen uptake in absolute values in normal-weight/non-obese and overweight/obese children. * *p* < 0.05; study’s first author name is presented in x axis; G = girls; B = boys; BMI = body mass index; BF = body fat percentage.

**Figure 2:**
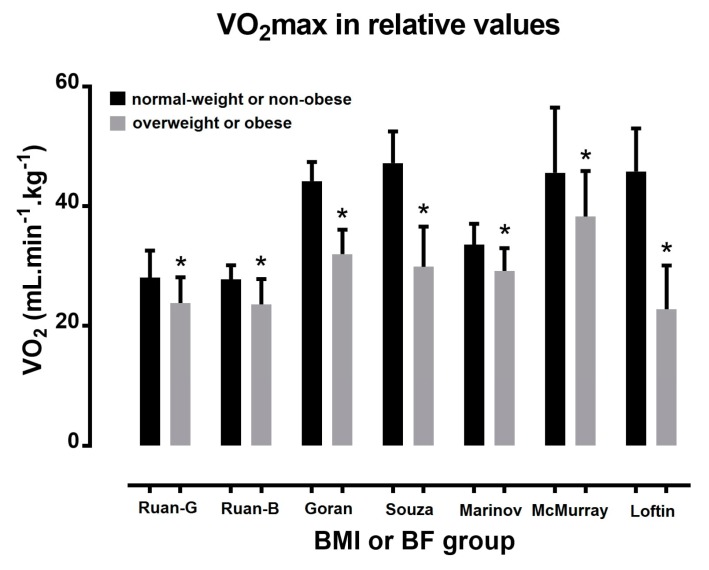
Maximal oxygen uptake in relative values in normal-weight/non-obese and overweight/obese children. * *p* < 0.05; study’s first author name is presented in x axis; G = girls; B = boys.

A large number of studies have shown higher absolute (L·min^−1^) (Figure 1) and lower relative (mL·kg^−1^·min^−1^) VO_2max_ (Figure 2) in overweight or obese children compared with their normal-weight peers [53,54,55]. For instance, Goran and colleagues [53], and Marinov and colleagues [54] observed higher absolute and lower relative VO_2max_ for obese than for non-obese children. Moreover, Ruan and colleagues [55] observed higher absolute and lower relative VO_2max_ for overweight or obese than for non-obese school children. In agreement with these studies, Pate and colleagues [19] recorded differences among overweight, at risk for overweight, and normal-weight groups; 41.6, 43.5, and 48.2 mL·kg^−1^·min^−1^ among boys, and 39.6, 37.6, and 35.9 mL·kg^−1^·min^−1^ among girls, respectively.

In the study of Souza and colleagues [21], although no statistical difference in absolute occurred between obese and normal-weight children, the former had lower relative than the latter. McMurray and colleagues [16] found higher VO_2max_ (L·min^−1^) for the overweight than for their normal-weight peers. Although Loftin and colleagues [56] showed similar values of VO_2max_ in L·min^−1^ between BMI groups, VO_2max_ in mL·kg^−1^·min^−1^ was 50% lower for obese girls than their normal-weight peers.

### 3.2. Relationship between Body Mass Index and Body Fat

The negative effect of BMI on VO_2max_ should be partially attributed to the relationship between BMI and BF. The magnitude of this relationship in children ranged from large to very large depending on sex and ethnicity [57,58], very large [59] to almost perfect [60]. This close affinity between BMI and BF might explain the abovementioned observation that overweight children have higher VO_2max_ in absolute values but lower in relative to BM values than their normal-weight peers. On the contrary, overweight have lower relative VO_2max_ as their body composition is less metabolically active since it consists of a higher BF.

### 3.3. Maximal Heart Rate and Heart Rate Acute Responses to Exercise Testing According to Body Mass Index

Overweight had lower HR_max_ than their normal-weight peers, both in a GXT on a cycle ergometer (186 vs. 196 bpm, respectively) or a walk/run test (175 vs. 197 bpm) in the study of Norman and colleagues [18] on 14–15 year-old children. Compared with non-obese, obese boys had higher acute HR response to Queen’s College step test (171 vs. 150 bpm) [34], and the same trend was noticed in girls who performed the same test (176 vs. 155 bpm) [33]. Maffeis and colleagues [28] found higher acute HR responses to walking and running on a treadmill in obese than in non-obese children (~9 years old); in addition, for a given exercise intensity, energy expenditure was also higher in obese than in non-obese, but it was comparable in the two groups when expressed in relation to BM values. On the contrary, Reybrouck and colleagues [22] found lower acute HR responses to exercise on a treadmill (i.e., normal vs. obese: 1st stage, 150 vs. 149 bpm; 2nd stage, 155 vs. 143 bpm; 3rd stage, 164 vs. 152 bpm; 4th stage, 172 vs. 162 bpm).

### 3.4. Heart Rate at Rest

HR_rest_ has been well discussed by many studies on overweight and obese children [18,20,24,61,62,63,64]. HR_rest_ in most of these studies has been measured in a seated position [18,24,26,62,63,65], in a few studies in a supine position [61,64], and position was not reported in one case [20]. HR recording usually lasted from 3 min [24] to 15 min [61]. The equipment to assess HR_rest_ includes electrocardiograph [18,64], blood pressure monitor [24,26,62,65], and HR monitor [61,63]. With regards to the role of BF, Govindan and colleagues [24] found that obese children had higher HR_rest_ than non-obese children among both boys (84.3 vs. 79.8 bpm, respectively) and girls (84.5 vs. 81.3 bpm). Charakida and colleagues [62] observed that overweight and obese children (10.5 years) had higher HR_rest_ than their normal-weight peers (72.4, 74.6 and 71.7 bpm, respectively). Norman and colleagues [18] found HR_rest_ to be higher in overweight (94 vs. 82 bpm) than in normal-weight children (14.5 years). Faria and colleagues [20] showed higher HR_rest_ in obese children than in normal-weight children for girls (78.1 vs. 74.6 bpm, respectively), but not for boys (73.3 vs. 73.6 bpm). There was only one study that did not record differences between overweight and normal-weight children for boys (81 vs. 80 bpm, respectively) or for girls (93 vs. 93 bpm) [65]. In addition to the abovementioned studies of cross-sectional design, another experimental approach was to examine the effect of an intervention exercise program on HR to exercise.

### 3.5. Sport Populations

Recent studies investigated differences in aerobic capacity between normal and overweight athletes [66,67,68,69,70,71]. Aerobic capacity was estimated by two tests: (*a*) physical working capacity test in HR 170 bpm (PWC_170_) conducted on a cycle ergometer, in which power relative to BM (W·kg^−1^) was recorded, and (*b*) a 3 min step test, in which acute HR responses were recorded at the end of the test and at the end of the first minute of recovery. In a study on male handball players (15 years), normal-weight had similar PWC_170_ (2.59 vs. 2.52 W·kg^−1^, respectively), HR at the end of the step test (140 vs. 145 bpm) and at the end of the first minute of recovery (96 vs. 99 bpm) than their overweight peers [70]. In research on male soccer players (12–21 years), normal-weight athletes had lower PWC_170_ in W and higher PWC_170_ in W·kg^−1^ than their overweight counterparts [69]. In addition, a study on female volleyball players (15 years) revealed similar PWC_170_ in W·kg^−1^ for normal-weight and overweight participants (1.97 vs. 1.84 W·kg^−1^, respectively) [67]. The protocol of PWC_170_ consisted of submaximal cycling against predetermined resistances for three 3 min stages aiming to elicit HR between 120 and 170 bpm. Thus, the performance in PWC_170_ was dependent of HR; i.e., the lower the HR response to a given exercise intensity, the higher the PWC_170_. Consequently, the relatively low performance of overweight athletes in PWC_170_ might reflect their relatively high acute HR responses.

### 3.6. Resting Metabolic Rate

RMR, usually measured by indirect calorimetry, is a major determinant of the daily total energy expenditure, and therefore, it is considered as a target-variable in any BM management program [72,73]. It has been observed that overweight boys had higher RMR than their normal-weight peers by 30% in a study where these two groups differed by 51% in BM [72]. A research on girls and boys reported 15% higher RMR and 45% higher BM in overweight children than their normal-weight peers [74]. It was also shown that overweight girls and boys had 11% higher RMR and 51% higher BM than their normal-weight peers [75]. Since RMR correlates with FFM and BM, RMR was disproportionately different from BM [75], the higher RMR in overweight children should be attributed mostly to their higher BM compared to their normal-weight peers.

### 3.7. Respiratory Quotient

Indirect calorimetry also provides information about the nutrients’ (carbohydrates, lipids, and protein) oxidation, which can be quantified using the RQ, i.e., the ratio of the carbon dioxide removed from the body divided by the oxygen consumed in the tissues [76]. Typical values of RQ range from 0.7 (lipid oxidation) to 1.0 (carbohydrate oxidation); thus, the estimation of RQ for a given exercise characterizes the substrate oxidation [30]. A comparison of BMI groups showed no difference in lipid oxidation during various submaximal loads of a GXT in prepubescent girls, but higher lipid oxidation in obese pubescent girls [77]. However, no difference in RQ was observed between normal-weight and overweight boys [30].

### 3.8. Limitations, Strengths, and Practical Applications

It should be highlighted that, although all ethnicities were considered during the search in the databases, it was acknowledged that most studies were conducted in Caucasian populations. With regards to ethnicity, differences have been observed in body composition and energy expenditure among ethnic groups (South Asian, East Asian, Southeast Asian, and Pacific Islanders) [78]. Moreover, BMI of Pacific Islanders was higher and their rate of physical growth more rapid compared to people of European descent living in the same country [79]. Thus, the findings of the present study should be generalized with caution to non-Caucasian ethnicities. In addition, it should be noted that maturation level was not considered in most of the existing research and this might also influence the outcome of the studies under examination. For instance, an elevated BMI has been associated with earlier puberty, especially in girls, and the relationship between BMI and onset of puberty might be influenced by biological (e.g., hormones) and environmental factors [80]. Most of the existing literature on acute HR responses to exercise among adults differing in BMI has obtained its data through maximal exercise testing, whereas most of the studies of cardiorespiratory fitness in BMI groups of children relied on field methods such as shuttle run endurance tests, and did not provide information about HR or VO_2_ variation [81,82]. Therefore, the need for studies comparing groups differing in BMI using submaximal exercise intensities, which characterize typical exercise training and longitudinal design was identified. The knowledge of acute cardiometabolic responses of various BMI groups to exercise testing might help practitioners to develop optimal exercise programs for BM management for overweight and obese children. In addition, our findings provided practical applications for practitioners engaged in exercise testing. Notably, caution is needed when interpreting VO_2max_ in the examined cohorts. Furthermore, it would be recommended for strength and conditioning coaches prescribing exercise that aerobic exercise should be less (in terms of duration and/or intensity) in overweight and obese children and adolescents than in their normal-weight peers in order to induce similar cardiometabolic acute responses. We were able to show that different measurement units (e.g., absolute versus relative to BM) influence the variation of VO_2max_ according to BMI. Furthermore, since overweight children achieved lower HR_max_ during GXT than their normal-weight peers, this should be considered in the evaluation of the indirect criteria used to assess the attainment of VO_2max_.

## 4. Conclusions

Compared with their normal-weight peers, a trend of higher HR_rest_ and HR during submaximal exercise and lower HR_max_ was observed among overweight and obese children. Independent from exercise mode (walking, running, cycling, or stepping), exercise was metabolically more demanding for obese and overweight children than for their normal-weight peers. Findings from this study are of practical relevance for practitioners working in the field of cardiorespiratory exercise testing in youth. Test data can be used to develop adequate training programs for BM management in youth. Based on the findings of this review, strength and conditioning coaches working with children and adolescents varying for BM and BF should be aware about the increased cardiometabolic cost induced by aerobic exercise in those being overweight or obese compared to their normal-weight peers.

## Abbreviations

BF	body fat percentage
bpm	beats per minute
DBP	diastolic blood pressure
FFM	fat-free mass
GXT	graded exercise test
HR	heart rate
HR_max_	maximal heart rate
HR_rest_	heart rate at rest
METs	metabolic equivalents
PE	Physical Education
PWC_170_	physical working capacity test in HR 170 bpm
RMR	resting metabolic rate
RQ	respiratory quotient
RPE	rate of perceived exertion
SBP	systolic blood pressure
VO_2_	oxygen uptake
VO_2max_	maximal oxygen uptake
